# Investigating the impact of ketogenic diets

**DOI:** 10.7554/eLife.103140

**Published:** 2024-10-18

**Authors:** Nalia Samba, Marie Gendrel

**Affiliations:** 1 Institut de Biologie de l’École Normale Supérieure, École Normale Supérieure, CNRS UMR 8197, INSERM U1024, Université Paris Sciences et Lettres Paris France

**Keywords:** ketone bodies, neurodevelopment, GABA, brain development, *C. elegans*

## Abstract

Exposure to ketone bodies in early development can reduce neurological impairments in a strain of the nematode *C. elegans* with PTEN defects.

**Related research article** Giunti S, Blanco MG, De Rosa MJ, Rayes D. 2024. The ketone body β-hydroxybutyrate ameliorates neurodevelopmental deficits in the GABAergic system of daf-18/PTEN *Caenorhabditis elegans* mutants. *eLife* **13**:RP94520. doi: 10.7554/eLife.94520.

For the past decade, many research efforts have focused on how nutrition can influence brain development and function. The ketogenic diet, for example, has emerged as a diet with promising therapeutic value. Relying on a low-carbohydrate, high-fat and moderate protein intake, it aims to replicate the metabolic effects of fasting by inducing a state of ketosis, in which the body produces and uses molecules known as ketone bodies as its energy source. This diet has been investigated as being potentially beneficial for neurodegenerative conditions such as Alzheimer’s and Parkinson’s disease, as well as for certain populations diagnosed with autism spectrum disorder (Al-Kuraishy et al., 2024; Tidman et al., 2024; Matthews and Adams, 2023). Interestingly, certain forms of autism spectrum disorder have also been frequently linked to a mutated version of an enzyme called PTEN (Yehia et al., 2020).

Widely studied in cancer research, PTEN is involved in the regulation of the cell cycle pathway – acting in part by triggering the activation of FOXO, a transcription factor that helps control how cells divide, die and use energy (Liu et al., 2022; Kotelevets et al., 2020). However, PTEN is also important in neurodevelopment, with defects in PTEN being associated with epilepsy, for instance (Skelton et al., 2020). One of the hallmarks of those neurodevelopmental diseases is aberrant activity of neural circuits; the proper function of these circuits relies on a strict balance between excitation and inhibition in the system, yet exactly how PTEN participates in this process has remained unclear so far. Now, in eLife, Sebastián Giunti, María Gabriela Blanco, María José De Rosa and Diego Rayes report findings that help clarify the link between PTEN, the balance between inhibitory and excitatory activity, and the neuroprotective impact of the ketogenic diet (Giunti et al., 2024).

To investigate these questions, the researchers (who are based at the Instituto de Investigaciones Bioquímicas de Bahía Blanca and the Universidad Nacional Del Sur) used the small worm *Caenorhabditis elegans* as a platform. They focused on its neuromuscular junction, where excitatory neurons that release acetylcholine cause muscles cells to contract, while inhibitory neurons producing GABA cause these fibers to relax. The powerful genetic tools available in *C. elegans,* combined with the fact that its nervous system (which is made up of 302 neurons and 7000 synapses) has been extensively mapped, make this organism an excellent model for studying the role of PTEN in detail (Corsi et al., 2015).

Giunti et al. performed a range of experiments comparing wild-type worms to animals in which the genes for PTEN, FOXO or both had been mutated. When exposed to drugs that exacerbate excitatory activity, the mutants were more likely to become paralysed. This hypersensitivity points to an imbalance in muscle activity, with an increase of muscle excitation over inhibition.

Next, the team monitored the escape response of the worms, such as their ability to quickly change direction by sharply turning their head and sliding it alongside their abdomen. Worms with defects in their inhibitory activity can rarely execute this movement as it requires the dorsal muscles to relax (which is under inhibitory control) while the ventral muscles hypercontract (due to excitatory activity); and indeed, both PTEN and FOXO mutants showed these defects.

To further investigate the role of these genes, Giunti et al. used light-based approaches to activate specific cells at will in the worms. In mutants, activating inhibitory neurons did not lead to the characteristic body elongation that normally takes place in wild-type animals due to their muscles relaxing; conversely, greater shortening of the body occurred when excitatory neurons were stimulated, likely due to fewer cells being in a relaxed state. These phenotypes are characteristic of defects in inhibitory neurotransmission, while also suggesting that excitatory activity is preserved. Additionally, the team monitored the morphology of neurons by specifically marking both inhibitory and excitatory cells with a fluorescent protein. In PTEN/FOXO mutants, only inhibitory neurons showed morphological alterations, with their axons failing to be targeted to muscles cells (Figure 1A). These experiments confirmed that inhibitory (or ‘GABA-ergic’) neurons displayed multiple developmental errors in PTEN/FOXO mutants, leading to altered function.

**Figure 1.**
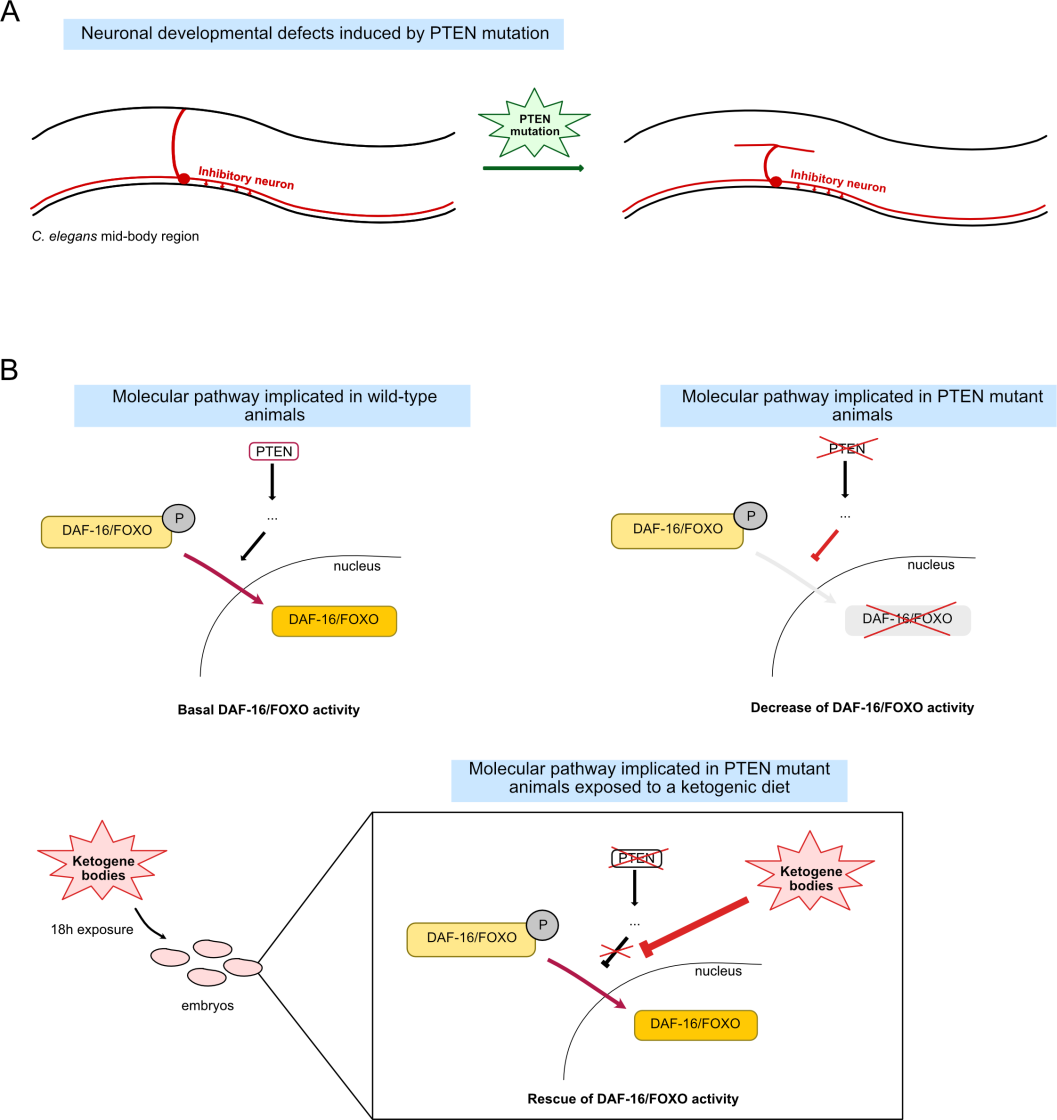
Linking PTEN, FOXO activity and the role of ketone bodies. (**A**) In the mid-body region of the microscopic worm *C. elegans*, a subset of excitatory (not shown) and inhibitory (red) motor neurons present in the ventral nerve cord (bottom) project onto the dorsal nerve cord (top). The absence of the gene coding for a protein known as PTEN leads to defects in how these inhibitory connections are established, resulting in various behavioural defects. (**B**) Under the influence of PTEN (top left), the transcription factor DAF-16 (equivalent to FOXO in humans) switches from an inactive, phosphorylated state (P; light yellow) to an activated form (dark yellow), which can be translocated to inside the nucleus. There, it can regulate the activity of genes important for cell function and development. The absence of PTEN prevents this relocation and leads to a decrease in FOXO activity (Ogg and Ruvkun, 1998; top right). Giunti et al. show that early exposure to ketone bodies (within the first 18 hours after egg laying, which includes a few hours after the worms hatch) is sufficient to rescue these effects and restore FOXO activity (bottom).

Finally, the team showed that defects in PTEN mutants could be alleviated by exposing the animals to ketone bodies early in their development. After this treatment, the mutants regained their ability to behave like wild-type worms, and the morphology and function of inhibitory cells were restored. To better understand the mechanisms at play, the team investigated whether these effects were mediated through FOXO activity by looking at the expression of a gene under the control of FOXO in various mutants. The results indicated that exposure to ketone bodies helped to counteract the loss of FOXO activity that stems from PTEN defects.

Overall, this work shows that PTEN is specifically involved in the development and function of inhibitory neurons, with this action being relayed at the level of the nucleus by FOXO. In the absence of PTEN, the reduction in FOXO activity can be mitigated by a ketogenic diet in early development (Figure 1B). As many neurotransmitters are conserved between *C. elegans* and vertebrates, such findings can provide insights into the biology of the human nervous system. In particular, they may shed a light on the link between autism spectrum disorder, PTEN mutations, and the impact of ketogenic diet on this condition. Many questions remain regarding the potential benefits of this and other diets on neurological function (Goya et al., 2020), how their effect is mediated, and what other molecular actors could be implicated.
